# Divergence in Gut Bacterial Community Among Life Stages of the Rainbow Stag Beetle *Phalacrognathus muelleri* (Coleptera: Lucanidae)

**DOI:** 10.3390/insects11100719

**Published:** 2020-10-21

**Authors:** Miaomiao Wang, Xingjia Xiang, Xia Wan

**Affiliations:** 1 School of Resources and Environmental Engineering, Anhui University, Hefei 230601, China; m_13228430417@163.com (M.W.); xjxiang@ahu.edu.cn (X.X.); 2 Anhui Province Key Laboratory of Wetland Ecosystem Protection and Restoration, Hefei 230601, China

**Keywords:** Lucanidae, gut bacteria, community, diet, life stages

## Abstract

**Simple Summary:**

*Phalacrognathus muelleri* is naturally distributed in Queensland (Australia) and New Guinea, and this species can be successfully bred under artificial conditions. In this study, we compared gut bacterial community structure among different life stages. There were dramatic shifts in gut bacterial community structure between larvae and adults, which was probably shaped by their diet. The significant differences between early instar and final instars larvae suggested that certain life stages are associated with a defined gut bacterial community. Our results contribute to a better understanding of the potential role of gut microbiota in a host’s growth and development, and the data will benefit stag beetle conservation in artificial feeding conditions.

**Abstract:**

Although stag beetles are popular saprophytic insects, there are few studies about their gut bacterial community. This study focused on the gut bacterial community structure of the rainbow stag beetle (i.e., *Phalacrognathus muelleri*) in its larvae (three instars) and adult stages, using high throughput sequencing (Illumina Miseq). Our aim was to compare the gut bacterial community structure among different life stages. The results revealed that bacterial alpha diversity increased from the 1st instar to the 3rd instar larvae. Adults showed the lowest gut bacterial alpha diversity. Bacterial community composition was significantly different between larvae and adults (*p* = 0.001), and 1st instar larvae (early instar) had significant differences with the 2nd (*p* *=* 0.007) and 3rd (*p* = 0.001) instar larvae (final instar). However, there was little difference in the bacterial community composition between the 2nd and 3rd instar larvae (*p* = 0.059). Our study demonstrated dramatic shifts in gut bacterial community structure between larvae and adults. Larvae fed on decaying wood and adults fed on beetle jelly, suggesting that diet is a crucial factor shaping the gut bacterial community structure. There were significant differences in bacterial community structure between early instar and final instars larvae, suggesting that certain life stages are associated with a defined gut bacterial community.

## 1. Introduction

Insects are the most diverse and abundant class of animals, living in multiple habitats, and feeding on various substrates [1,2]. The guts of insects are colonized by diverse microorganisms that play integral roles in their hosts, including affecting the hosts’ metabolism, providing essential amino acids, vitamins and nitrogen for the host, promoting efficient digestion of nutrient-poor diets and recalcitrant foods, aiding the defense and detoxification ability and protecting hosts from potentially harmful microbes [3]. Associations between microorganisms and insect hosts are widespread in nature. Studies on Cerambycidae species revealed that their gut bacterial community is dominated by diet type, developmental stage and gut compartment [4]. Insect gut microorganisms are recognized to originate from their environment and diet [5]. Beetles (Coleoptera) are the most diverse group of insects [6]. Almost all beetles undergo holometabolism, including a pupal stage compared with incomplete metamorphosis. The study of *Agrilus mali* (Coleoptera: Buprestidae) and *Popillia japonica* (Coleoptera: Scarabaeidae) has suggested that the metabolic activity of the intestine decreases in the pupal stage and morphology changes during the insects’ metamorphosis influence the associated bacteria communities [3,7]. Moreover, larvae and adults feed on different foods, thus, occupying distinct ecological niches in the carbon cycle of forest ecosystems [8]. The guts of beetles are excellent models to study the variety of gut microorganisms that contribute to hosts’ digestion, detoxification, development, pathogen resistance and physiology [1,2,5,9].

Previous studies have focused on the gut bacterial community of coleopteran pests to advance progress in pest control [10,11], including invasive bark beetles (Scolytidae) and longhorn beetles (Cerambycidae), that mainly feed on tree xylem and phloem. These species are considered to be serious pests of forests, as they grow and develop while feeding on lignocellulose [12,13]. Many studies have investigated gut microbial species that are critical for the development and survival of the hosts [14,15]. The aim of these studies was to identify how to damage and alter the core microbiota to cause the death of pests, thereby developing new pest control strategies [16,17,18,19]. Further, gut bacteria in these pests contributed to understand the degrading functions by digesting cellulose and lignin (i.e., wood, litter, and humus) to promote host growth [10,11,12,20,21]. 

In addition, some beetle gut microbial studies have involved dung beetles (Scarabaeidae) and burying beetles (Silphidae). Adult dung beetles feed primarily on nutritionally rich dung particles, while larvae consume coarser dung particles with a higher C/N ratio [22]. There are studies comparing the gut microbiota differences between larvae and adults [22], suggesting that diet is a factor in the composition of gut microbiota. Individual studies have focused on maternal transmission [23] to explore the similarity of gut microbes between larvae and adult females or males [24]. Burying beetles use small vertebrate carcasses as a source of food for their developing larvae [25]. Studies of their gut microflora have focused on the functions of the gut microbiota, such as the digestion of carrion and the detoxification of microbial toxins [26,27].

There is little information about the gut bacterial community of stag beetles, although they are one of the most important beetles in forests. The larval stages live in and feed on decaying wood, and the adults utilize fermented tree sap and over–ripe fruits [28]. To date, studies of microorganisms associated with stag beetles involve fungi, particularly those transported externally on the exoskeleton or in specialized structures known as mycangia [29,30,31,32]. Fungi in the larval gut could contribute to forest matter cycling through turning a low nutrition food source into available nutrients [32,33]. Likewise, little work has been done on the gut bacteria of stag beetles using Illumina MiSeq high-throughput sequencing, with the exception of a study by Jiang [34] who analyzed the gut bacterial structure in *Odontolabis fallaciosa* adults. However, little work has focused on gut bacterial communities of *Phalacrognathus muelleri* (Coleptera: Lucanidae) during successive life stages with different diets.

This study represents the first comprehensive analysis of the gut bacterial community of the rainbow stag beetle *P. muelleri* during different life stages using high- throughput sequencing. This species is naturally distributed in Queensland (Australia) and New Guinea. The beetle has become a very popular pet due to the colorful body and interesting male mandibles and is successfully bred under artificial conditions in many countries. We investigated 1st, 2nd, and 3rd instar larvae and adults that were raised under standard rearing conditions (see Materials and Methods). Specifically, the main goal of this study was to explore the differences of gut bacterial community composition and diversity during successive life stages of *P. muelleri* with different diets.

## 2. Materials and Methods 

### 2.1. Sample Collection and Rearing Conditions

All the samples of *P. muelleri* were obtained from the Mu–Ye Insect Company (Lishui, Zhejiang, China), including the 1st instar (L1, eight individuals), the 2nd instar (L2, twelve individuals), the 3rd instar (L3, nine males and nine females) and the adults (six males and ten females) (Table S1). These samples were artificially reared under constant conditions (temperature: 18–22 °C). Larvae were reared in pudding boxes containing decaying wood with a 50% water content; the larvae were frequently observed as to feeding status, and the feed was regularly replaced and continual1y rehydrated. Adults were reared on beetle jelly and their feed was regularly replaced. 

### 2.2. Sample Dissection

Before dissection, all samples were disinfected for 3 min with 70% ethanol, and then rinsed with distilled water to ensure no contamination on the body surface [35,36]. Beetles were transported to a horizontal clean bench, and dissected in 10–fold diluted phosphate–buffered solution (PBS) (500 mL, NaCl 1.37 M, KCL 26.8 mM, Na_2_HPO_4_ 81.0 mM, KH_2_PO_4_ 17.6 mM, pH 7.2–7.4) [37] under a stereomicroscope. The midgut and hindgut were removed using sterile fine tip forceps and placed into 2 mL Lysing Matrix E under sterile conditions to avoid contamination. In addition, 1st instar, 2nd instar, 3rd instar and adults were dissected at five days, ten days, ten days, and ten days, respectively.

### 2.3. Microbial DNA Extraction and PCR Amplification 

DNA extractions were performed on each gut sample using QIAamp FAST DNA Stool Mini Kits (Qiagen Inc. Valencia, CA, USA) according to the operating instructions. The extracted DNA was dissolved in 75 μL of elution buffer, quantified by NanoDrop ND–1000 (Thermo Scientific, Wilmington, DE, USA) and stored at −20 °C. The purified DNA of each sample served as an amplification template. The primer sets 515F (5′-GTGCCAGCMGCCGCGG-3′) and 907R (5′-GGACTACHVGGGT WTCTAAT-3′) [38], were used to amplify the V4 to V5 variable regions of the bacterial 16S rRNA gene fragments for the Illumina Mi–Seq platform (PE 250) at Majorbio (Shanghai, China). The PCR reactions were carried out in 20 μL reaction mixtures containing 4 μL 5 × FastPfu Buffer, 2 μL 2.5 mM dNTPs, 0.8 μL 5 uM of each primer, 0.4 μL DNA polymerase, 0.2 μL BSA, 10 ng template DNA and deionized μltrapure water (to 20 μL). The PCR conditions were as follow: initial at 95 °C (3 min) follows by 27 cycles at 95 °C (30 s), 55 °C (30 s), and 72 °C (45 s), and final extension at 72 °C for 10 min. To check for contamination, PCR negative controls were performed without added DNA template. Negative PCR controls did not contain detectable PCR product and were not processed for sequencing. Triplicate reaction mixtures per sample were pooled together and purified using an agarose gel DNA purification kit (TaKaRa, Bio Inc., Kusatsu, Japan). The PCR products were pooled in equimolar amounts (10 pg for each sample) before sequencing.

### 2.4. Processing of Sequence Data

The raw data were processed by the Quantitative Insights Into Microbial Ecology (QIIME v.1.9 software [39]. Low–quality sequences (below an average quality score of 30 and the length < 250 bp) were deleted. High–quality sequences were clustered into Operational Taxonomic Units (OTUs; 97% similarity; de novo approach). The chimeras were eliminated by the USEARCH (V.1.8.0). The ribosome database project classifier selects the most abundant sequence in each OTU as the representative sequence [40], which was aligned by PyNAST [39]. In order to perform similar sequencing and homogenization among samples, we used randomly selected subsets of 26,000 sequences (lowest sequence read depth; repetition with 20 times) per sample to compare bacterial community composition and diversity for all samples [41].

### 2.5. Statistical Analysis

The differences in alpha diversity and the relative abundance of dominant phyla among life stages were based on one-way analysis of variance (ANOVA) with Tukey Honestly Significant Difference (HSD) Post-Hoc testing (SPSS 20.0 for Windows, Chicago, IL, USA) [41]. Linear discriminant analysis (LDA) effect size (LEfSe) was used to identify intestinal bacterial taxa with significant differences among host stages. This method uses the non–parametric Kruskal–Wallis test with default settings (an alpha value of 0.05 and an effect size threshold of 2) in a rank sum test to identify biomarkers [42]. The differences in bacterial community composition between different stages were analyzed by non–metric multidimensional scaling (NMDS) and analysis of similarity (ANOSIM; permutations = 999) using the vegan package (Version 2.0–10 [43] in R v.2.8.1 [44,45]. The contribution of bacterial OTUs (operational taxonomic units) to the differences between stages was analyzed by SIMPER using the vegan package in R software. Indicator analysis was performed using the labdsv package in R software [46]. Bacterial diversity and the relative abundance of dominant phyla were analyzed by one-way ANOVA.

### 2.6. Data Availability Statement

The raw data were submitted to the Sequence Read Archive (SRA) of NCBI under the accession number SUB8060438.

## 3. Results

### 3.1. Intestinal Bacterial Alpha Diversity

**Figure 1:**
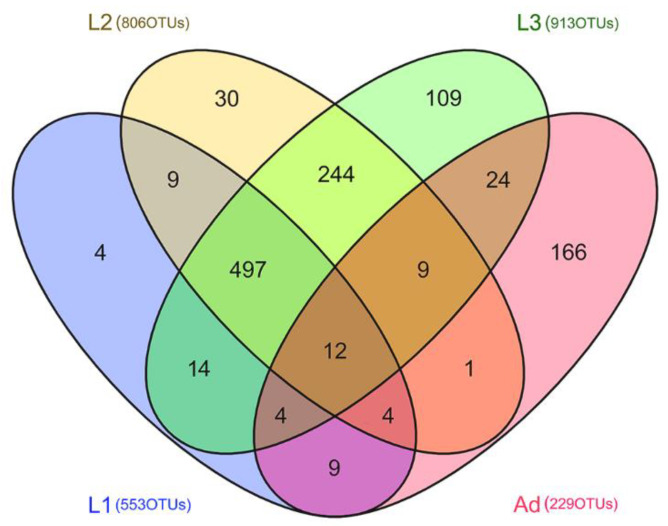
Venn diagram showing the co-occurence of the OTUs among samples from different stages. Numbers in parentheses indicate total OTUs in each stage group, and numbers inside the Venn diagram indicate unique and shared OTUs. OTU, operational taxonomic unit.

In this study, a total of 1,997,394 quality–filtered bacterial sequences were retrieved from the 54 samples for the primer pair F515/R907, ranging from 26,163 to 61,214 sequences per sample (Table S2). A total of 1136 bacterial OTUs were detected (relative abundance (%) <0.01%), with all samples ranging from 200 to 5912 (97% similarity), and 12 OTUs were shared across all life stages. There were 553, 806, 913 and 229 OTUs in L1, 1st instar; L2, 2nd instar; L3, 3rd instar and Ad, Adults, respectively. In addition, there were unique OTUs within each stages, especially for adults, where the unique OTUs accounted for 72.4% with the 229 distinct OTUs observed, and the unique OTUs of 1st instar, 2nd instar, 3rd instar accounted for 0.7%, 3.7% and 11.9%, separately (Figure 1).

**Figure 2:**
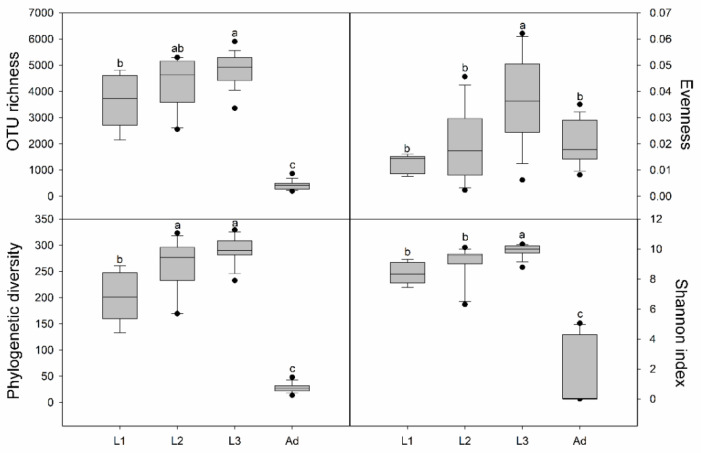
Intestinal bacterial alpha-diversity in different developmental stages. The bottom and top of the box denote the first and third quartiles respectively; the band inside the box denotes median; error bars denote standard deviations; different letters above bars represent significant differences from Tukey’s HSD comparisons (*p* < 0.05). L1, 1st instar; L2, 2nd instar; L3, 3rd instar; Ad, Adults.

Bacterial alpha diversity indicators (i.e., OTU richness, Shannon index, evenness, and phylogenetic diversity) were calculated at a depth of 26,000 randomly selected sequences per sample. According to the index estimation results, the bacterial alpha diversity of the larvae was higher than that of the adults, and the bacterial diversity of larvae increased with successive instars (Figure 2). In different sexes alpha diversity analysis, with the exception of OTU richness, which was relatively higher in females than in males of L3, all other indicators showed little differences in L3. There were no significant differences in the alpha diversity of adults between females and males (Figure S1). 

**Figure 3:**
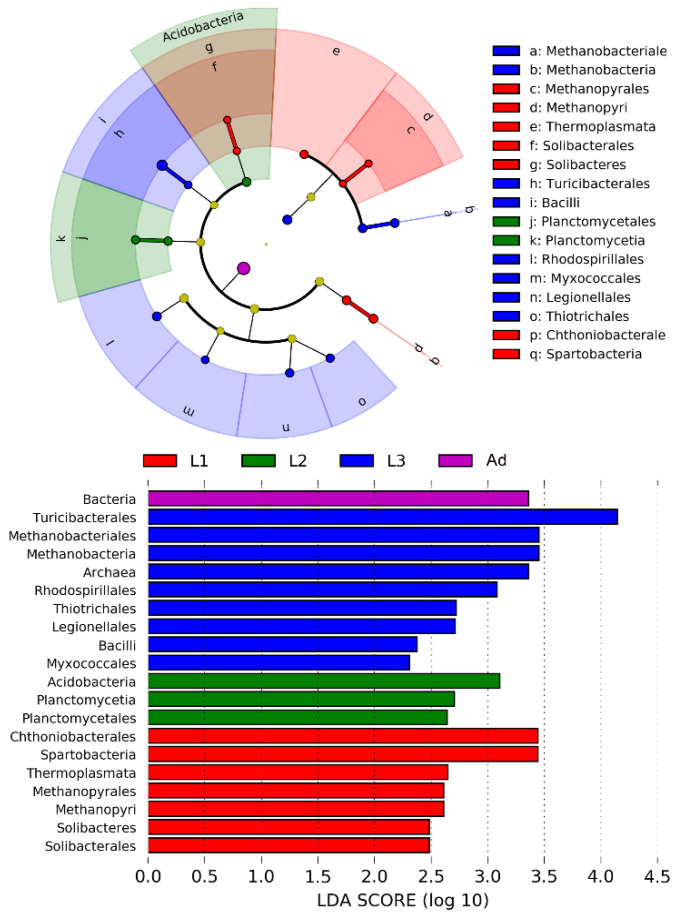
LEfSe analysis of intestinal bacterial biomarkers associated with host types. Identified phylotype biomarkers ranked by effect size, and the alpha value was < 0.05. Cladogram representing the taxonomic hierarchical structure of the phylotype biomarkers identified among four host types, red, phylotypes overrepresented in gut of L1; green, phylotypes statistically overrepresented in gut of L2; blue, phylotypes statistically overrepresented in gut of L3; purple, phylotypes statistically overrepresented in gut of Ad. L1, 1st instar; L2, 2nd instar; L3, 3rd instar; Ad, Adults.

**Figure 4:**
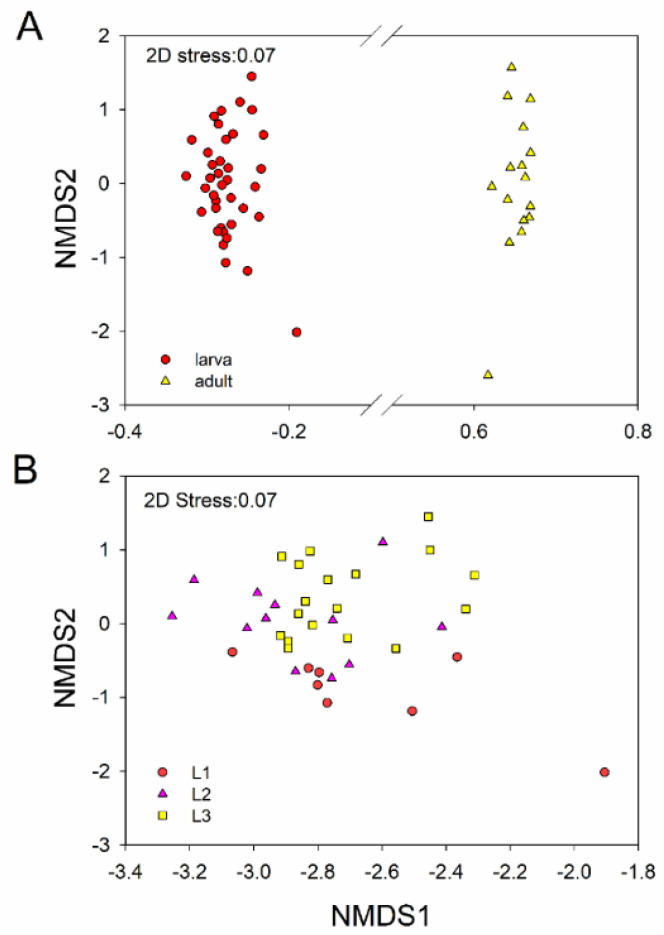
Non-metric multidimensional scaling (NMDS) plot showing bacterial community composition between larvae and adults (**A**), and contrasts between different larval instars (**B**). L1, 1st instar; L2, 2nd instar; L3, 3rd instar; Ad, Adults.

LEfSe analysis further identified specific bacterial taxa that were differentially abundant across all life stages. The results showed that bacteria in four classes (i.e., Spartobacteria, Solibacteres, Thermoplasmata, Methanopyri) and three orders (i.e., Chthoniobacterale, Solibacterales, Methanopyrales) were significantly more abundant in L1. Bacteria in two phyla (i.e., Planctomycetales, Acidobacteria) and one class (i.e., Planctomycetia) were significantly more abundant in L2. Bacteria from two classes (i.e., Bacilli, Methanobacteri) and five orders (i.e., Myxococcales, Thiotrichales, Rhodospirillales, Methanobacteriales, Legionellalesi) were significantly more abundant in the gut of L3 (Figure 3). SIMPER analysis revealed that OTU_46981 (*Sporomusa*; 2.91%), OTU_63703 (Ruminococcaceae; 2.14%) made primary contributions to community differences between L1 and L2; OTU_46981 (*Sporomusa*; 2.94%) and OTU_63703 (Ruminococcaceae; 2.17%) were the main OTUs responsible for the differences between L1 and L3; OTU_46981 (*Sporomusa*; 2.89%) and OTU_38714 (*Dysgonomonas*; 7.72%) contributed to differences in the bacterial community composition between L1 and Ad; OTU_46981 (*Sporomusa*; 0.80%) and OTU_9882 (*Dysgonomonas*; 1.74%) produced the significant differences in composition between L2 vs. L3; OTU_9882 (*Dysgonomonas*; 1.60%) and OTU_38714 (*Dysgonomonas*; 7.71%) were the principal OTUs contributing to differences between L2 and Ad, OTU_38714 (*Dysgonomonas*; 7.72%) and OTU_53348 (Lactobacillales; 4.04%), contributed to the differences in bacterial community composition between L3 and Ad (Table S3). Indicator analysis was used to identify bacterial OTUs that were specifically associated with various life stages. There were different indicator species in the L1, L2, L3 and Ad (Table S4), and some indicators were different in females and males (Table S5). We also compared the bacterial community composition across different life stages using NMDS analysis; significantly different communities were present in larvae and adults (ANOSIM: *p* = 0.001, Table 1; Figure 4). And 1st instar larvae (early instar) had significant differences with the 2nd (*p =* 0.007) and 3rd (*p* = 0.001) instar larvae (final instar) (Table 1; Figure 4).

### 3.2. Intestinal Bacterial Community Structure

**Figure 5:**
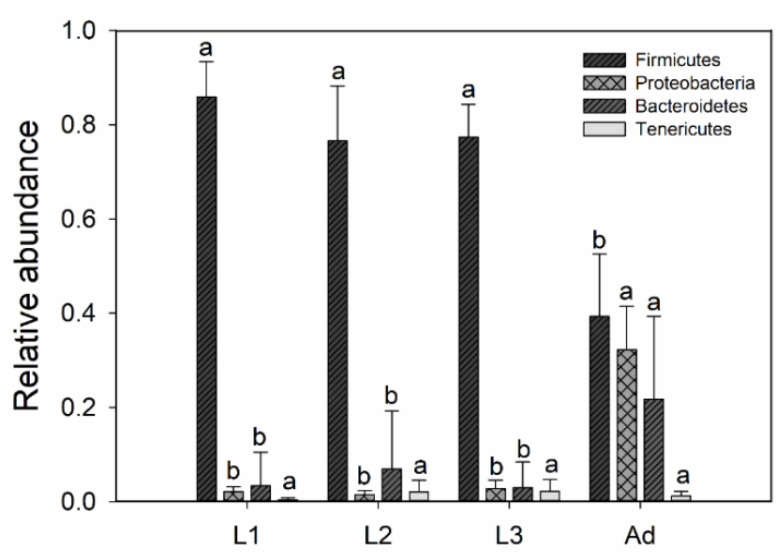
Relative abundance of bacterial taxa at phylum in the gut of different life stages of *P. muelleri*. Bars represent mean; error bars denote standard deviation, letters above bars represents significant differences from one-way ANOVA (*p* < 0.05). L1, 1st instar; L2, 2nd instar; L3, 3rd instar; Ad, Adults.

The dominant intestinal bacterial phyla across all samples were Firmicutes (74.72%), Proteobacteria (12.67%), Bacteroidetes (10.81%) and Tenericutes (1.80%) (Figure 5). These dominant phyla had significant differences in relative abundance in larvae and adults except for Tenericutes (one–way ANOVA: *p* < 0.05), and there were no significant differences among the three larval instars (one–way ANOVA: *p* > 0.05). The relative abundance of Firmicutes showed a significant decrease, and the relative abundances of Proteobacteria, and Bacteroidetes showed significant increases in adults relative to larvae (one–way ANOVA: *p* < 0.05) (Figure 5). There were no significant differences in dominant phyla between females and males of L3. Proteobacteria showed significant differences between male and female adults, with a higher relative abundance in males (Figure S2). 

## 4. Discussion

We investigated the gut bacterial community of *P. muelleri* across different life stages under artificial breeding conditions through Illumina MiSeq high-throughput sequencing. This is the first study of the gut bacteria of *P. muelleri* focusing on different life stages. Four phyla—Firmicutes, Proteobacteria, Bacteroidetes and Tenericutes—were predominant in the gut bacteria across all stages, which is consistent with prior studies in other beetles (i.e., bark beetles, longhorn beetles, herbivorous beetles and burying beetles) [12,13,26,47]. Firmicutes and Proteobacteria were the dominant phyla in *Anoplophora glabripennis* [12], *Hylobius abietis* [48], *Monochamus alternatus* and *Psacothea hilaris* [4], *Nicrophorus vespilloides* [26], and *Popillia japonica* [7]. Firmicutes, Proteobacteria and Bacteroidetes were also the dominant phyla in *Holotrichia parallela* [47], *Dendroctonus valens* [13], and *Dendroctonus rhizophagus* [10], and Tenericutes also was the dominant phylum in *Odontolabis fallaciosa* (Lucanidae) [34]. Prior research demonstrated that intestinal Firmicutes play significant role in the degradation of complex plant carbohydrates. Bacteroides species are able to degrade diverse plant polysaccharides [49], thereby improving the host’s ability to digest food. Proteobacteria contribute to nitrogen fixation and food metabolism to keep the host healthy [50]. The aggregation of these communities in insect gut is dependent on physicochemical conditions of gut compartments [5], such as the available oxygen and pH [4].

In our study, the larval alpha diversity was significantly higher than that of adults (Figure 2), in consistent with a previous study found in species of *Melolontha hippocastani* [51]. Intestinal bacteria communities are considered as originate from the environment and diet [3]. Thus, the high diversity displayed in larvae may due to the vast microbial colonized via specific nutritional complementation from different diet [50,52]. Moreover, adults rarely feed after emergence, even if enough beetle jelly is given. Among the three larval instars of *P. muelleri*, the alpha diversity increased in successive instars, a result in accordance with gut bacteria in the *Melolontha hippocastani*.

According to the NMDS analysis and ANOSIM analysis, gut bacterial community composition was significantly different between larvae and adults (ANOSIM: *p* = 0.001, Table 1, Figure 4). During the growth of *P. muelleri*, the dominant phyla of Firmicutes decreased its relative abundance, while the relative abundance of Bacteroidetes and Proteobacteria were increased in adults (Figure 5). Previous studies have demonstrated that gut bacteria communities can be influenced by host diet [14,21,35,53]. Among the three larval instars of *P. muelleri*, their gut bacterial structures reflected significant divergence. Gut bacterial community composition was significantly different and between L1 and L3 (ANOSIM: *p* = 0.001, Figure 4) and between L1 and L2 (ANOSIM: *p* = 0.007, Figure 4), while the difference was relatively small between L2 and L3 (ANOSIM: *p* = 0.059, Figure 4). We inferred that the compositional differences were related to food intake. Chen [54] also suggested that the physiological and biochemical conditions within the larval alimentary tract affect bacterial community structure. Different food intake and content among three instars could affect the intestinal microbial composition, as in scarab beetle larvae [55]. Relatively speaking, as hosts grew, the food intake increased.

Notably, understanding the function among intestinal bacteria is important to explore the complexity host development process. We observed Ruminococcaceae, Veillonellaceae, Christensenellaceae and Lachnospiraceae was dominated in the larval stages (Table S4), three families that were previously observed to co-occur in the bacterial gut microbiota of the longhorned beetle and bark beetle larvae [54]. They can help their host degrade the decaying wood and metabolize cellulose to promote larval digestion. Such as Ruminococcaceae are vital to degradation of lignocellulose [56]. Christensenellaceae and Lachnospiraceae are important for the degradation of plant material and cellulose catabolism [49,57], and Christensenellaceae is related to host health [58]. Interestingly, the family Veillonellaceae was considered as a probiotic providing benefits for energy balance and producing various volatile fatty acids to lower gut pH [59], which is conducive to host gut fitness. Enterobacteriaceae was dominanted in the adults (Table S5), in consistent with a previous study found in bark beetle [37]. Bacteria in this family can ferment glucose, reduce nitrates to nitrites, and degrade sucrose to provide nutrients for hosts [60].

Likewise, bacteria in the five genera *Sporomusa*, *Candidatus*, *Coprococcus*, *Prevotella* and *Turicibacter* were isolated from larvae (Table S4). These groups contribute to external immunity and food fermentation [5,61]. Members of genera *Dysgonomonas*, *Enterococcus* are abundant in the adult stages, similar in the *Odontolabis fallacios* adults [34], *Lactococcus*, *Trabulsiella*, *Neorickettsia*, *casseliflavus*, *Selenomonas* were also found in the termites gut [61]. They were primarily involved in lignocellulose decomposition, food fermentation and enhancement of metabolic capacity of the host [4,62,63]. This information illustrated that different diets cause differences in gut bacteria, a result that has been reported in the scarab beetle, *Popillia japonica* [7], in the fruit fly *Drosophila suzukiiis* and in the pine weevil *Hylobius abietis* [52,64].

We further considered the influence of gender on intestinal bacteria. In L3, there was a barely significant difference between males and females (ANOSIM: *p* = 0.047). Adults also differed little between males and females (ANOSIM: *p* = 0.578), supporting similar findings in bark beetles from earlier studies [13,21]. This suggested that male and female individuals subjected to standard rearing conditions harbored communities that were highly conserved in structure and membership. We suspect that this may be due to: (i) the beetles being bred in captivity, having a homogeneous diet, and subject to the same living conditions; (ii) the beetles were raised alone with no opportunistic communication. Both factors can affect colonization by gut microbiota [65]. In addition, *P. muelleri* in our study had not yet attained sexual maturity (this usually takes 30 to 60 days, while our sampling was done at 10 days after emergence), and this may have affected microbial colonization.

## 5. Conclusions

Our study revealed the structure of the gut bacterial community in different life stages of the rainbow stag beetle, *P. muelleri*. Results showed that bacterial community composition was significantly different between larvae and adults, and between early instar and final instar larvae. Diet and life stages can thus collectively influence the gut bacterial community composition. This research provides a basis for subsequent studies on the roles of these intestinal bacteria in stag beetle development and ecology. Results also contribute to a better understanding of the potential role of gut microbiota in a host’s growth and development, and the data will benefit stag beetle conservation in artificial feeding conditions. However, there were certain limitations in this research, as the level of biological replication was relatively low. In addition, we did not sequence the intestinal bacterial in pupae. These limitations should be addressed in future studies. In addition, future research will develop more complex bioinformatics tools on the basis of high-throughput sequencing to analyze the undiscovered microorganisms in the insect intestines, and gain insight into their functions for their hosts.

## Figures and Tables

**Table 1:** Differences in bacteria community composition depending on different development stages and sexes by analyses of similarities (ANOSIM).

Stages	ANOSIM	
	*R*	*p*
L1 vs. L2	0.302	0.007
L1 vs. L3	0.497	0.001
L1 vs. Ad	0.999	0.001
L2 vs. L3	0.109	0.059
L2 vs. Ad	1.000	0.001
L3 vs. Ad	1.000	0.001
L3♀ VS L3♂	0.200	0.047
Ad♀ VS Ad♂	−0.040	0.578

L1, 1st instar; L2, 2nd instar; L3, 3rd instar; Ad, Adults. ♀, female; ♂ male.

## Supplementary Materials

The following are available online at https://www.mdpi.com/2075-4450/11/10/719/s1, Figure S1. Intestinal bacterial alpha-diversity in different sexes, Figure S2. Relative abundance of bacterial taxa at the phylum level in the guts of different sexes of *P. muelleri*, Table S1. All sample materials of *Phalacrognathus Muelleri*, after removing outlier sample values, Table S2. Gut bacterial sequences across the samples, Table S3. SIMPER analysis showing the contribution of bacteria OTUs to the differences in bacterial community composition in the gut depending on different life stages, Table S4. Indicator species in different life stages, Table S5. Indicator species in different sexes, Table S6. The total OTU table.
