# Supplementary material for: Divergence in Gut Bacterial Community Among Life Stages of the Rainbow Stag Beetle Phalacrognathus muelleri (Coleptera: Lucanidae)
Source: Insects. 2020 Oct 21;11(10):719. doi: 10.3390/insects11100719 (PMC7589407; doi:10.3390/insects11100719)
Supplement: Supplementary file 1 [file insects-11-00719-s001.pdf]

Divergence in gut bacterial community among life stages of the rainbow stag beetle *Phalacrognathus muelleri* (Coleptera: Lucanidae)

Miaomiao Wang [1,2,†], Xingjia Xiang [1,2,†] and Xia Wan [1,2,*]

[1] School of Resources and Environmental Engineering, Anhui University, Hefei 230601, China; m_13228430417@163.com (M.W.); xjxiang@ahu.edu.cn (X.X.);

[2] Anhui Province Key Laboratory of Wetland Ecosystem Protection and Restoration, Hefei 230601, China.

* Correspondence: wanxia@ahu.edu.cn

† These authors contributed equally to this article.

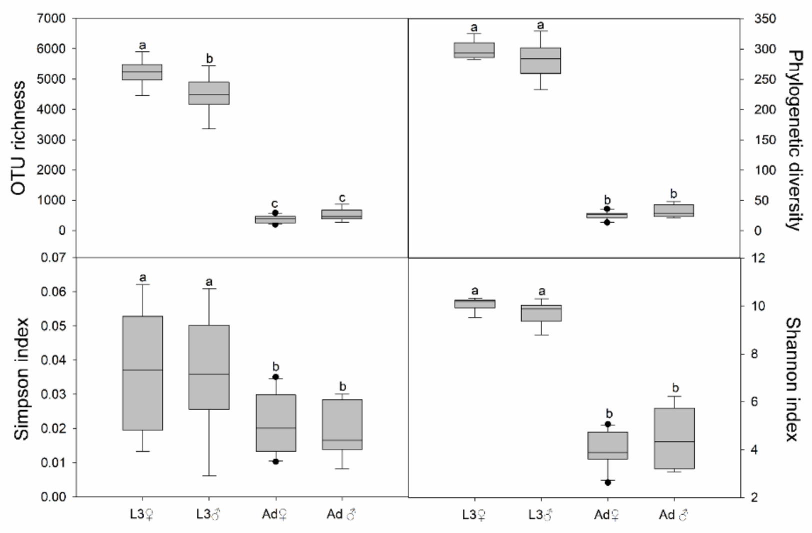

Figure S1. Intestinal bacterial alpha-diversity in different sexes. The bottom and top of the box denote the first and third quartiles, respectively; the band inside the box denotes the median; error bars denote standard deviations; different letters above bars represent significant differences from Tukey's HSD comparisons ($P < 0.05$).

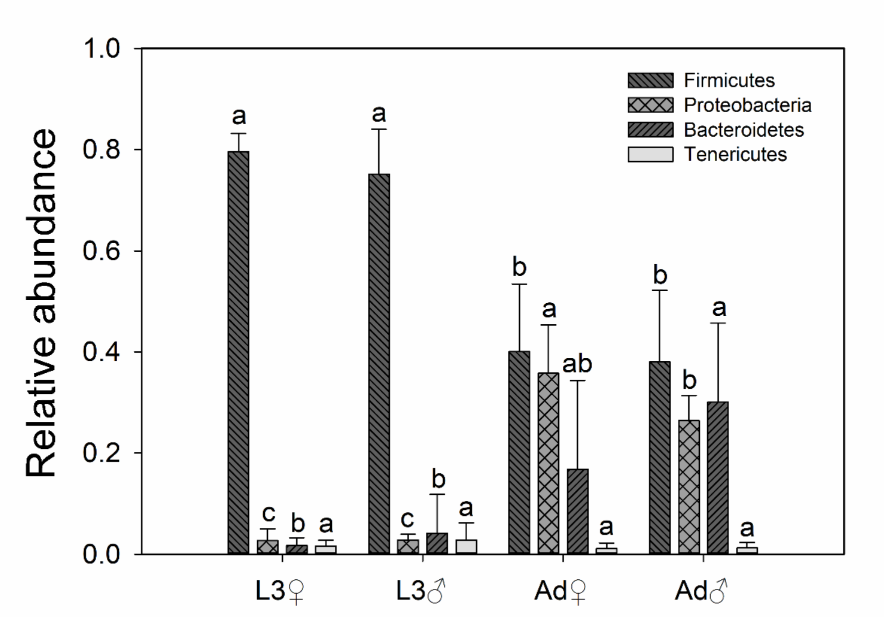

Figure S2. Relative abundance of bacterial taxa at the phylum level in the guts of different sexes of *P. muelleri*. The bottom and top of the box denote the first and third quartiles respectively; the band inside the box denotes median; error bars denote standard deviations; different letters above bars represent significant differences from Tukey's HSD comparisons ($P < 0.05$). L3,3rd female; Ad, Adults; ♀ female, ♂, male.

Table S1. All sample materials of *Phalacrognathus Muelleri*, after removing outlier sample values.

Phalacrognathus muelleri	Number	Artificial feeding conditions
Adults-female	10	
Adults-male	6	The larvae fed on fermented wood chips, with a
3rd instar-female	9	moisture content of about 55%, and the adults fed on
3rd instar-male	9	beetle jelly. The best breeding temperature is 18–22℃.
2nd instar	12	
1st instar	8	

10,6,9,9,12,8 represents the number of beetles in each stage.

Table S2. Gut bacterial sequences across the samples.

Sample	Sequences Bacteria	OTU-richness	Evenness	Phylogenetic diversity	Shanonn
pha1	29673	3503.2	0.010	189.56	8.208
pha2	36430	2696.4	0.015	151.44	7.812
pha3	36859	4565.3	0.008	261.33	8.480
pha4	30217	4820.4	0.015	249.76	9.330
pha5	35961	3983.2	0.014	212.49	8.769
pha6	40006	4635.5	0.016	241.14	9.212
pha7	32011	2766.5	0.008	186.06	7.724
pha8	60787	2160.7	0.015	133.54	7.455
pha9	31814	5277.3	0.035	305.81	10.112
pha10	34380	4585.0	0.030	276.66	9.577
pha11	33717	4455.9	0.016	233.13	9.083
pha12	33756	4707.5	0.029	294.33	9.729
pha13	36861	5302.4	0.019	281.57	9.684
pha14	37581	5175.8	0.022	270.19	9.673
pha15	39506	4348.1	0.006	278.03	9.063
pha16	36095	5120.2	0.014	323.55	9.623
pha17	33834	3343.5	0.046	232.60	9.012
pha18	32762	2556.6	0.006	169.67	6.990
pha19	39560	4962.0	0.015	296.34	9.639
pha20	39127	2734.8	0.002	171.20	6.312
pha21	30612	5439.8	0.026	283.81	9.882
pha22	31727	5080.4	0.047	293.10	10.200
pha23	26193	5906.9	0.013	325.49	10.079
pha24	37706	5525.4	0.037	288.04	10.211
pha25	46267	5244.8	0.062	311.83	10.329
pha26	35618	4455.1	0.013	283.24	9.516
pha27	44946	4862.6	0.036	291.03	9.960

pha28	40781	5248.4	0.050	308.12	10.254
pha29	38875	5218.2	0.055	308.67	10.259
pha30	34989	4812.7	0.031	289.75	10.040
pha31	30947	4762.5	0.049	302.67	10.056
pha32	28145	4189.6	0.061	272.73	9.876
pha33	33067	4351.2	0.036	247.50	9.841
pha34	36097	4471.7	0.044	283.67	9.941
pha35	39726	5440.2	0.052	329.43	10.315
pha36	42321	4974.8	0.006	302.10	9.178
pha37	34628	4134.7	0.032	278.33	9.534
pha38	29080	3363.0	0.021	232.92	8.793
pha39	32243	385.7	0.029	27.52	4.680
pha40	36489	433.7	0.010	28.56	3.580
pha41	37618	453.8	0.019	25.69	4.460
pha42	29850	307.0	0.022	22.46	3.838
pha43	31216	524.0	0.023	31.77	4.921
pha44	37560	581.8	0.016	36.16	5.058
pha45	33942	242.9	0.012	21.61	2.621
pha46	34815	247.0	0.031	19.79	3.901
pha47	33558	201.0	0.035	13.81	3.616
pha48	37217	383.0	0.014	27.47	3.857
pha49	50726	452.3	0.017	24.99	4.341
pha50	61214	278.1	0.016	21.24	3.066
pha51	35285	419.4	0.008	25.28	3.264
pha52	35933	455.3	0.016	32.57	4.305
pha53	53668	865.0	0.030	48.14	6.227
pha54	43398	602.3	0.028	41.07	5.563

pha, phalacrognathus muelleri.

Table S3. SIMPER analysis showing the contribution of bacteria OTUs to the differences in bacterial community composition in the gut depending on different life stages. Taxonomic leaves: o, order; f, family; g, genus; s, species.

OTU	Taxa	Contribution (%)					
		L1 vs L2	L1 VS L3	L1 VS Ad	L2 VS L3	L2 VS Ad	L3 VS Ad
46981	g_*Sporomusa*	2.91	2.94	2.89	0.80	—	—
63703	f_Ruminococcaceae	2.14	2.17	—	—	—	—
9882	g_*Dysgonomonas*	1.85	—	—	1.74	1.60	—
64289	f_Ruminococcaceae	1.85	1.85	—	—	—	—
63013	f_Ruminococcaceae	1.60	1.63	—	—	—	—
22575	f_Ruminococcaceae	—	1.36	—	—	—	—
38714	g_*Dysgonomonas*	—	—	7.72	—	7.71	7.72
53348	o_Lactobacillales	—	—	4.04	—	4.04	4.04
16920	f_Enterococcaceae	—	—	2.25	—	2.25	—
56864	f_Enterobacteriaceae	—	—	2.25	—	2.25	2.25
3991	f_Ruminococcaceae	—	—	—	1.03	—	—
41803	g_*Dysgonomonas*	—	—	—	1.03	—	—
51020	f_Ruminococcaceae	—	—	—	0.89	—	—
16920	g_*Enterococcus*	—	—	—	—	—	2.25
53432	g_*Enterococcus*	—	—	—	—	—	1.60

L1, 1st instar; L2, 2nd instar; L3, 3rd instar; Ad, Adults.

Table S4. Indicator species in different life stages. Taxonomic leaves: p, phylum; o, order; f, family; g, genus; s, species.

Age	Indicator species	Taxonomy	*P*	Relative abundance (%)
L1	OTU46981	g__*Sporomusa*	0.001	1.380
	OTU63703	f__Ruminococcaceae	0.001	1.010
	OTU64289	f__Ruminococcaceae	0.001	0.796
	OTU63013	f__Ruminococcaceae	0.001	0.752
	OTU39327	f__Veillonellaceae	0.004	0.527
	OTU22575	f__Ruminococcaceae	0.001	0.374
	OTU2880	f__Lachnospiraceae	0.001	0.314
	OTU59017	f__Lachnospiraceae	0.001	0.255
	OTU64163	f__Ruminococcaceae	0.008	0.185
	OTU54096	o__Clostridiales	0.001	0.181
	OTU22369	o__Clostridiales	0.009	0.178
	OTU45631	f__Ruminococcaceae	0.001	0.151
	OTU59433	o__Clostridiales	0.001	0.143
	OTU17749	f__Christensenellaceae	0.002	0.136
	OTU22431	f__Veillonellaceae	0.040	0.117
	OTU43374	g__*Candidatus* s_*Xiphinematobacter*	0.031	0.107
	OTU57033	g__*Coprococcus*	0.015	0.106
L2	OTU3991	f__Ruminococcaceae	0.002	0.002
	OTU56399	f__Ruminococcaceae	0.043	0.043
	OTU62347	f__Ruminococcaceae	0.003	0.003
	OTU7266	f__Veillonellaceae	0.011	0.011
L3	OTU54440	o__Clostridiales	0.047	0.236
	OTU40364	f__Ruminococcaceae	0.011	0.190
	OTU46566	g__*Prevotella*	0.017	0.132
	OTU53750	f__Ruminococcaceae	0.035	0.118
	OTU12535	f__Christensenellaceae	0.011	0.108
	OTU20496	p__Tenericutes	0.004	0.105
	OTU33251	g__*Turicibacter*	0.019	0.105
Ad	OTU38714	g__*Dysgonomonas*	0.001	4.570
	OTU53348	o__Lactobacillales	0.001	2.390
	OTU16920	g__*Enterococcus*	0.001	1.330
	OTU56864	f__Enterobacteriaceae	0.001	1.330
	OTU53432	g__*Enterococcus*	0.004	0.943
	OTU22437	g__*Lactococcus*	0.001	0.889
	OTU37829	f__Enterobacteriaceae	0.001	0.719

OTU56978	g__*Trabulsiella*; s__*farmeri*	0.001	0.658
OTU6013	g__*Neorickettsia*	0.001	0.621
OTU28724	g__*Dysgonomonas*	0.01	0.565
OTU22440	o__Pasteurellales	0.001	0.503
OTU56171	f__Spirochaetaceae	0.001	0.459
OTU1011	g__*Enterococcus*	0.002	0.414
OTU10350	f__Enterobacteriaceae	0.008	0.410
OTU2298	g__*Enterococcus*	0.024	0.379
OTU11298	g__Enterococcus s__*casseliflavus*	0.001	0.360
OTU21712	g__*Dysgonomonas*	0.004	0.347
OTU56564	f__Enterobacteriaceae	0.035	0.341
OTU6148	g__*Enterococcus*	0.003	0.308
OTU22423	g__*Dysgonomonas*	0.005	0.251
OTU22434	g__Dysgonomonas	0.028	0.251
OTU9685	g__*Trabulsiella*; s__*farmeri*	0.001	0.245
OTU56505	f__Enterobacteriaceae	0.001	0.226
OTU933	g__*Enterococcus*	0.005	0.218
OTU19711	g__*Enterococcus*	0.001	0.204
OTU53514	o__Pasteurellales	0.001	0.184
OTU61281	g__*Lactococcus*	0.001	0.179
OTU25877	f__Enterobacteriaceae	0.01	0.176
OTU55930	g__*Trabulsiella*; s__*farmeri*	0.001	0.173
OTU55229	f__Enterobacteriaceae	0.003	0.161
OTU21311	p__Tenericutes	0.001	0.141
OTU43367	p__Tenericutes	0.004	0.136
OTU24969	g__*Trabulsiella*; s__*farmeri*	0.003	0.125
OTU53191	f__Enterobacteriaceae	0.001	0.125
OTU54956	f__Enterobacteriaceae	0.001	0.114
OTU47524	g__*Enterococcus*	0.011	0.106
OTU20888	g__*Selenomonas*	0.003	0.101

L1, 1st instar; L2, 2nd instar; L3, 3rd instar; Ad, Adults.

Table S5. Indicator species in different sexes. Taxonomic leaves: p, phylum; c, class; f, family; g, genus; s, species.

Age	Indicator species	Taxonomy	*P*	Relative abundance (%)
L3♀	OTU59017	f__Lachnospiraceae	0.024	0.255
	OTU45631	f__Ruminococcaceae	0.017	0.151
	OTU17749	f__Christensenellaceae	0.019	0.136
	OTU12535	f__Christensenellaceae	0.034	0.107
	OTU7266	f__Veillonellaceae	0.037	0.104
	OTU61323	f__Ruminococcaceae	0.010	0.101
	OTU47622	f__Ruminococcaceae	0.023	0.083
	OTU27998	f__Veillonellaceae	0.030	0.082
	OTU18152	f__Veillonellaceae	0.030	0.081
	OTU44853	g__*Sporomusa*	0.050	0.078
	OTU64052	f__Ruminococcaceae	0.006	0.070
L3♂	OTU30174	f__Lachnospiraceae	0.050	0.052
	OTU18618	o__Clostridiales	0.005	0.051
Ad♀	OTU28724	g__*Dysgonomonas*	0.018	0.565
	OTU53514	o__Pasteurellales	0.033	0.184
	OTU25626	g__*Dysgonomonas*	0.039	0.015
	OTU27484	o__Pasteurellales	0.004	0.014
	OTU37474	g__*Trabulsiella*	0.03	0.013
	OTU32338	g__*Enterococcus*	0.006	0.009
	OTU21328	g__*Lactococcus*	0.02	0.009
	OTU5616	o__Lactobacillales	0.028	0.007
	OTU49107	g__*Enterococcus*	0.029	0.006

L3♀, 3rd female; L3♂ 3rd male; Ad♀, Adults female.

Table S6. The total OTU table. L1, 1st instar; L2, 2nd instar; L3, 3rd instar; Ad, Adults, ♀, female;♂ male.

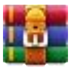

OTU.rar
